# Associations of airway inflammation and responsiveness markers in non asthmatic subjects at start of apprenticeship

**DOI:** 10.1186/1471-2466-10-37

**Published:** 2010-07-06

**Authors:** Valérie Demange, Pascal Wild, Denis Zmirou-Navier, Paul Tossa, Abraham Bohadana, Annick Barbaud, Christophe Paris

**Affiliations:** 1 Department of Epidemiology, INRS, Rue du Morvan, Vandœuvre-lès-Nancy, 54500, France; 2 INSERM U954, School of Medicine, 9, avenue de la forêt de Haye, Vandoeuvre-lès-Nancy, 54500, France; 3 Nancy University Medical School, France; 4 Ecole des Hautes Etudes en Santé Publique (EHESP), Rennes, France; 5 Nancy Hospital poison centre, Central Hospital, University Hospital of Nancy, Nancy, France; 6 Service of Pneumology, Brabois Adults Hospital, Vandoeuvre-lès-Nancy, France; 7 Department of Dermatology, Fournier Hospital, University Hospital of Nancy, Nancy, France

## Abstract

**Background:**

Bronchial Hyperresponsiveness (BHR) is considered a hallmark of asthma. Other methods are helpful in epidemiological respiratory health studies including Fractional Exhaled Nitric Oxide (FENO) and Eosinophils Percentage (EP) in nasal lavage fluid measuring markers for airway inflammation along with the Forced Oscillatory Technique measuring Airway resistance (AR). Can their outcomes discriminate profiles of respiratory health in healthy subjects starting apprenticeship in occupations with a risk of asthma?

**Methods:**

Rhinoconjunctivitis, asthma-like symptoms, FEV1 and AR post-Methacholine Bronchial Challenge (MBC) test results, FENO measurements and EP were all investigated in apprentice bakers, pastry-makers and hairdressers not suffering from asthma. Multiple Correspondence Analysis (MCA) was simultaneously conducted in relation to these groups and this generated a synthetic partition (EI). Associations between groups of subjects based on BHR and EI respectively, as well as risk factors, symptoms and investigations were also assessed.

**Results:**

Among the 441 apprentice subjects, 45 (10%) declared rhinoconjunctivitis-like symptoms, 18 (4%) declared asthma-like symptoms and 26 (6%) suffered from BHR. The mean increase in AR post-MBC test was 21% (sd = 20.8%). The median of FENO values was 12.6 ppb (2.6-132 range). Twenty-six subjects (6.7%) had EP exceeding 14%. BHR was associated with atopy (p < 0.01) and highest FENO values (p = 0.09). EI identified 39 subjects with eosinophilic inflammation (highest values of FENO and eosinophils), which was associated with BHR and atopy.

**Conclusions:**

Are any of the identified markers predictive of increased inflammatory responsiveness or of development of symptoms caused by occupational exposures? Analysis of population follow-up will attempt to answer this question.

## Background

There are many tests for investigating Occupational and non-Occupational asthma (OA). Traditionally, attention has been focused on airway obstruction indicators such as Forced Expiratory Volume in 1 second (FEV1), Airway Resistance (AR) and measurement of non-specific Bronchial Hyperresponsiveness (BHR). More recently, airway inflammation indicators have gained acceptance, including induced sputum cell counts or Fractional Exhaled Nitric Oxide (FENO)[1].

In patients with mild to moderate asthma, FENO has been found to correlate with BHR, reversibility of airway obstruction, serum Eosinophil Cationic Protein (ECP), blood eosinophilia[2] and AR parameters post methacholine[3]. Among adolescents in clinical remission and without treatment for atopic asthma, FENO has been also correlated with BHR[4]. In patients with rhinitis, a relationship has been found between sputum eosinophil numbers[5,6] or eosinophil numbers in a nasal smear[7] or sputum ECP level[6] and BHR.

Associations between airway responsiveness and inflammation markers have therefore been widely studied in the clinical field and have confirmed their clinical significance in relation to diagnosing disease or monitoring drug responsiveness[1,8,9]. In a recent publication, Olin et al. have shown that increased FENO can predict new-onset wheeze in a general population[10]. However, little is known concerning the helpfulness of these markers in a healthy population exposed to airborne hazardous substances that may trigger OA.

This paper presents baseline descriptive data on respiratory health and operation in non-asthmatic apprentices recently recruited for training in occupations subject to asthma risk. In particular, we have tried to identify cross-sectionally fragile subgroups on inclusion in a cohort[11], based on associations of 1) BHR (the gold standard in terms of obstructive disorder investigation) and, 2) post-methacholine AR, FENO, eosinophils percentages in nasal lavage fluid and symptoms. We have considered gender, training history, atopy and smoking habits.

## Methods

The protocol has been detailed previously[11]. Briefly, all apprentice bakers, pastry-makers and hairdressers starting training programmes at six vocational schools in Lorraine, North-Eastern France, were invited to take part in research. Those who accepted were included, provided they had neither a history of previous occupational exposure to substances known to induce OA nor a history of physician-diagnosed asthma. Three visits followed the initial inclusion visit. The study was approved by the local ethical committee and written consent was obtained from the apprentices or their parents, depending on the apprentices' ages. Only data from the first visit were used for this study.

### Questionnaire

Symptoms were assessed using a standard questionnaire, which covered personal and demographic information, past chest diseases and symptoms, and past and present smoking habits[12].

Hay fever was defined as sneezing, itchy, runny or stuffy nose occurring almost every day or from time to time in spring or summer.

Clinical atopy was defined as hay fever and/or eczema and/or asthma in childhood and atopy in family as asthma or allergy in siblings and/or parents.

Rhinoconjunctivitis-like symptoms were defined as itchy, runny, stuffy nose or sneezing and/or red, burning or weeping eyes, except during a respiratory infection occurring irregularly almost every day or from time to time. Asthma-like symptoms were defined as wheezing, chest tightness, shortness of breath or coughing, except during a respiratory infection or under exercise conditions.

### Fractional Exhaled Nitric Oxide (FENO)

FENO was measured according to ATS/ERS recommendations[9] using a chemiluminescence analyzer (NIOX^® ^2.0 system; Aerocrine AB, Solna, Sweden).

FENO was expressed as observed parts per billion (ppb) and as a percentage of predicted values based on gender, tobacco usage status and personal atopy[13].

### Pulmonary Function Tests

Pulmonary function tests were undertaken after measurement of FENO (SensorMedics Corporation, Datalink, Montpellier, France). At least three baseline forced expiratory manoeuvres meeting recommended criteria[14] were recorded and largest forced vital capacity (FVC) and FEV_1 _values were recorded for analysis. A mean oscillatory resistance of 4 to 16 Hz (Rrs4-16) was used as outcome variable.

Non-specific airway responsiveness was assessed using the methacholine bronchial challenge (MBC) test based on the procedure described above[15]. Three cumulative doses of methacholine (100, 600 and 1600 μg respectively) were administered in succession[11]. The test was finished after the last dose of methacholine or when the FEV_1 _fell by 20% or more below the baseline value. In this case, the subjects were considered as MBC+ 20%. The difference between the maximum of Rrs4-16, after methacholine inhalation increase, and baseline Rrs4-16 divided by baseline Rrs4-16 was defined as the increase in resistance post-MBC test.

### Eosinophil Count in Nasal Lavage Fluid

Nasal lavage was performed using the Hilding's method[16]. Slides with >30% squamous cells were rejected. Absolute cells were counted and eosinophils percentages were calculated (EP).

### Skin Prick Tests

Skin Prick Tests (SPT) were performed using dust mites, animal danders, pollens and moulds (Stallergenes Laboratories, Fresnes, France; ALK-Allerbio Laboratory, Varennes en Argonne, France). A positive SPT was defined as a wheal diameter 3 mm or more larger than those obtained with the negative controls after 20 minutes[11]. Personal atopy was defined as the presence of a positive response to at least one common allergen[17].

### Statistical Analyses

Statistical analyses were conducted using the Stata package (Stata, College Station, TX, USA).

In order to study *a priori *links between inflammation markers and hyperresponsiveness in this young healthy population at the start of their occupational exposure, multiple correspondence analysis (MCA) and hierarchical classifications were therefore conducted to discriminate groups of subjects. Outcome variables were those characterising BHR; i.e. FEV1 fall and increase in Rrs4-16 post-MBC test; airway inflammation; FENO (in predicted percentages); EP; rhinoconjunctivitis- and asthma-like symptoms. Quantitative variables were converted into three classes of qualitative variable using the following distribution of the post-MBC test fall of FEV1, i.e. MBC+ 20% (26 highest values of fall and higher than 20%), MBC+ 15% (33 next highest values and higher than 15%) and no fall (371 last values). For EP, the three classes were divided into the 26 highest values and the next 25 values because only 51 values involved a detectable number of eosinophils.

We then studied the differences between subjects with and without BHR (defined as MBC+ 20%) based on gender, training history, tobacco usage status and atopy (in family, clinical and personal) and on post-MBC test Rrs4-16 increase, FENO and EP. We conducted the same analysis with the subject groups.

## Results

Four hundred and forty one apprentices were included, representing 24% of the 1839 apprentices invited to participate[11] (Table 1). Bakers and pastry-makers represented more than 60% of the group. Roughly half of these subjects were non smokers with fewer female than male smokers.

**Table 1:** Characteristics of the apprentice cohort at baseline (% (n), mean [sd]).

	Males	Females	Total
n	56.9% (251)	43.1% (190)	441
Training track			
Bakery	60.6% (152)	4.7% (9)	36.5% (161)
Pastry	34.7% (87)	12.6% (24)	25.2% (111)
Hairdressing	4.8% (12)	82.6% (157)	38.3% (169)
Age (years)	16.8 [1.1]	17.1 [1.7]	16.9 [1.4]
Body Mass Index	22.1 [4.1]	21.9 [3.7]	22.0 [3.9]
Tobacco consumption			
Current smokers	51.4% (129)	41.1% (78)	46.9% (207)
Age at starting smoking	14.0 [1.6]	13.7 [1.8]	13.9 [1.7]
Pack years	1.7 [1.6]	1.7 [1.6]	1.7 [1.6]
Past smokers	2.0% (5)	4.2% (8)	3.0% (13)
Non-smokers	46.6% (117)	54.7% (104)	50.1% (221)
Atopy in family*	32.7% (82)	40.0% (76)	35.8% (158)
Clinical atopy†	20.7% (52)	20.5% (39)	20.6% (91)
Personal atopy‡ (97 missing data)	35.7% (70)	25.7% (38)	31.4% (108)
Baseline spirometry§			
Missing data (% (n))	1.6% (4)	1.1% (2)	1.4% (6)
FEV1	91.4 [11.0]	93.6 [9.5]	92.3 [10.4]
FVC	90.7 [12.0]	92.0 [10.6]	91.3 [11.4]
FEV1/FVC (%)	101.3 [8.4]	102.0 [7.0]	101.6 [7.9]
Baseline airway resistance (kPa.L.s^-1^)	0.25 [0.07]	0.29 [0.06]	0.27 [0.07]
MBC test			
Airways responsiveness			
Missing data (% (n))	3.2% (8)	1.6% (3)	2.5% (11)
MBC+ >20%	6.6% (16)	5.3% (10)	6.0% (26)
MBC+ 15 to 20%	8.2% (20)	7.0% (13)	7.7% (33)
Airway resistance			
Rrs4-16 increase (%)||			
>54.6%	7.4% (18)	4.5% (8)	6.2% (26)
>42.3% and <54.6%	9.1% (22)	6.2% (11)	7.8% (33)
<42.3%	83.5% (202)	89.4% (160)	86.0% (362)
Clinical indicators of airway inflammation			
Rhinoconjunctivitis-like symptoms¶	8.8% (22)	12.1% (23)	10.2% (45)
Asthma-like symptoms#	3.2% (8)	5.3% (10)	4.1% (18)
Quantitative indicators of airway inflammation			
FENO in percent predicted**			
>225%	6.6% (15)	6.8% (11)	6.7% (26)
>140% and <225%	8.7% (20)	8.0% (13)	8.4% (33)
<140%	84.7% (194)	85.2% (138)	84.9% (332)
Eosinophil count in nasal fluid lavage			
Missing data (% (n))	13.5% (34)	8.9% (17)	11.6% (51)
>1%	6.5% (14)	6.4% (11)	6.4% (25)
>14%	8.3% (18)	4.6% (8)	6.7% (26)

Rrs4-16: mean oscillatory resistance from 4 to 16 Hz; MBC Methacholine Bronchial Challenge test.

* asthma or allergy in siblings and/or parents; † hay fever and/or eczema in childhood and/or asthma in childhood; ‡ defined as a positive response to at least one common allergen at the Skin Prick Tests

§ in percent predicted according to the European Respiratory Society

|| increase = 100.(maximum of Rrs after methacholine inhalation - baseline Rrs)/baseline Rrs

¶ in case of itchy, runny, stuffy nose, or sneezes and/or about red, burning or weeping eyes, excepted during a respiratory infection

# in case of wheezing, chest tightness, shortness of breath, or cough, excepted during a respiratory infection or under exercise condition;** according to gender, tobacco usage status and personal atopy ([13])

Atopy in family was more often declared by females than by males (40% versus 33%), whereas the reverse was found for personal atopy (36% versus 26%). SPT could not be applied to the first 74 subjects for technical reasons.

BHR prevalence was identical in both genders, as was symptom prevalence (approximately 10% for rhinoconjunctivitis-like symptoms and 6% for asthma-like symptoms). Only one subject declared asthma-like symptoms and was MBC+ 20% without positive SPT. The mean increase in the AR post-MBC test was 21% (sd = 20.8%) (data not shown).

The median of FENO values was 12.6 ppb [2.6-132 range] (data not shown). Predicted mean FENO values were approximately 30% lower than expected[13]. However, the relationship between predicted FENO with personal atopy persisted, showing that the FENO on atopy adjustment did not fully correct its effect [Additional file 1]. Distribution of absolute FENO values and FENO in percent predicted according to sex, tobacco usage status and personal atopy is shown [Additional file 2].

The prevalence of high EP was almost twice as great in males as in females (Table 1). The EP of 26 subjects (6.7%) exceeded 14%.

The two axes produced by MCA and hierarchical classification explain 70% of the variance and allow us to distinguish four groups of items [Additional file 3]. The highest FENO and EP values lie to the left of the horizontal axis (group 1), whilst highest BHR level and EP mid-value lie on the right of this axis (group 2). In relation to the vertical axis, presence of symptoms and highest resistances lie above the axis (group 3), whilst FENO, EP and highest BHR level lie below the axis. Absence of symptoms or lowest test values lie at the origin of both axes and have no discriminating effect (group 4). The BHR mid-level has no discriminating effect on the vertical axis. The horizontal axis appears to agree closely with inflammation markers, whilst the vertical axis agrees closely with presence of symptoms and AR.

A second hierarchical subject classification based on proximity to the four item groups only identified two subject groups because of the small numbers of subjects defined by the group 2 and 3 items. These were therefore regrouped with the group 1 items. The widest group comprised 302 subjects without symptoms and/or the lowest outcome values. The second group comprised 39 subjects mostly defined by highest FENO or EP values. These two markers were strongly and positively associated (p < 0.001) (Table 2). We therefore called this group "positive eosinophilic inflammation" (EI+) and the widest group "negative eosinophilic inflammation" (EI-).

**Table 2:** Association between FENO and eosinophils percentages in nasal lavage fluid as qualitative variables (percentages in column) (N = 341).

	FENO in percent predicted according to Travers*		
Percentages of eosinophils	<140%	>140% and <225%	>225%
<1%	88.5% (261)	84.0% (21)	57.1% (12)
>1% and <14%	7.5% (22)	0% (0)	4.8% (1)
>14%	4.1% (12)	16.0% (4)	38.1% (8)

*according to gender, tobacco usage status and personal atopy ([13])

Personal atopy only reached statistical significance, when considering apprentices with respect to BHR (Table 3). BHR at inclusion was unrelated to gender, training history, tobacco usage status and predicted FEV1 percentage. Atopy in family was more frequent in subjects with, rather than without, BHR (p = 0.19). More than 60% of subjects with BHR were atopic compared to 28% without BHR. High values of FENO and EP were more often observed in the BHR group, but this relationship did not reach statistical significance.

**Table 3:** Associations between MBC test outcome and constitutional, behavioural characteristics and markers of airway responsiveness and inflammation (percentage in column (n)) (N = 341).

	MBC- FEV1's fall <20% (N = 322)	MBC FEV1's fall ≥20% (N = 19)	p
Constitutional characteristics			
Gender: Male	56.2% (181)	52.6% (10)	0.76
Atopy in family*	37.6% (121)	52.6% (10)	0.19
Clinical atopy†	19.9% (64)	10.5% (2)	0.55**
Personal atopy‡ (61 missing data)	27.4% (72)	64.7% (11)	<0.01**
Behavioural characteristics			
Training track			
Bakery	36.3% (117)	26.3% (5)	
Pastry making	25.5% (82)	26.3% (5)	0.69**
Hairdressing	38.2% (123)	47.4% (9)	
Tobacco usage status			
Non smoker	49.4% (159)	42.1% (8)	
Current smoker	47.2% (152)	52.6% (10)	0.54**
Past smoker	3.4% (11)	5.3% (1)	
Baseline FEV1§			
>80.8%	86.3% (278)	79.0% (15)	
>76.0% and <80.7%	8.7% (28)	10.5% (2)	0.31**
<76.0%	5.0% (16)	10.5% (2)	
Rrs4-16 increase post MBC test (%) (mean (sd))			
<42.4%	87.3% (281)	84.2% (16)	
>42.4% and <54.6%	6.8% (22)	10.5% (2)	0.75**
>54.6%	5.9% (19)	5.3% (1)	
Clinical indicators of airway inflammation			
Rhinoconjunctivitis-like symptoms||	9.6% (31)	5.3% (1)	1.0**
Asthma-like symptoms¶	4.0% (13)	5.3% (1)	0.56**
Quantitative indicators of airway inflammation			
FENO in ppb (median (first-third quartiles)	12.4 (8.9-17.6)	16.4 (10.3-35.0)	0.19††
FENO in percent predicted according to Travers#			
<140%	87.3% (281)	73.7% (14)	
>140% and <225%	7.1% (23)	10.5% (2)	0.09**
>225%	5.6% (18)	15.8% (3)	
Eosinophil count in nasal fluid lavage			
<1%	86.7% (279)	79.0% (15)	
>1% and <14%	6.5% (21)	10.5% (2)	0.37**
>14%	6.8% (22)	10.5% (2)	

Rrs4-16: mean oscillatory resistance from 4 to 16 Hz; MBC: Methacholine Bronchial Challenge test.

* asthma or allergy in siblings and/or parents; † hay fever and/or eczema in childhood and/or asthma in childhood; ‡ defined as a positive response to at least one common allergen at the Skin Prick Tests

§ in percent predicted according to the European Respiratory Society

|| in case of itchy, runny, stuffy nose, or sneezes and/or about red, burning or weeping eyes, excepted during a respiratory infection; ¶ in case of wheezing, chest tightness, shortness of breath, or cough, excepted during a respiratory infection or under exercise condition.

# according to gender, tobacco usage status and personal atopy ([13])

** Fisher's exact test; †† Mann-Whitney test

There was no difference between groups EI+ and EI- regarding gender, training history, tobacco usage status and predicted FEV1 percentage (Table 4). There were more than twice cases with personal atopy in the EI+ group than in EI- group. More specifically, personal atopy was strongly associated with predicted FENO percentage (p < 0.001), but not with EP (p = 0.15) [Additional file 1]. Similarly, BHR prevalence was twice as great in the EI+ group than in the EI- group. The highest increases in Rrs4-16 were more often observed in the EI+ group, but the difference was not statistically significant. This was the same for symptoms. The EI+ group had higher values of FENO and this was statistically significant. Similar associations were found for the highest EP values.

**Table 4:** Associations between groups without (EI-) and with eosinophilic inflammation (EI+) and constitutional, behavioural characteristics and markers of airway responsiveness and inflammation (percentage in column (n)) (N = 341).

	Group EI- (N = 302)	Group EI+ (N = 39)	p
Constitutional characteristics			
Gender: Male	56.3% (170)	53.9% (21)	0.77
Atopy in family*	37.1% (112)	48.7% (19)	0.16
Clinical atopy†	19.9% (60)	15.4% (6)	0.67**
Personal atopy‡ (61 missing data)	25.7% (64)	61.3% (19)	<0.001
Behavioural characteristics			
Training track			
Bakery	36.8% (111)	28.2% (11)	
Pastry making	23.8% (72)	38.5% (15)	0.14
Hairdressing	39.4% (119)	33.3% (13)	
Tobacco usage status			
Non smoker	48.0% (145)	56.4% (22)	
Current smoker	48.7% (147)	38.5% (15)	0.36**
Past smoker	3.3% (10)	5.1% (2)	
Baseline FEV1§			
>80.8%	86.1% (260)	84.6% (33)	
>76.0% and <80.7%	8.9% (27)	7.7% (3)	0.70**
<76.0%	5.0% (15)	7.7% (3)	
MBC test			
Airways responsiveness			
MBC-	88.1% (266)	69.2% (27)	
MBC+ ≥15 and <20% (% (n))	7.3% (22)	18.0% (7)	<0.01**
MBC+ ≥20% (% (n))	4.6% (14)	12.8% (5)	
Rrs4-16 increase (%) (mean (sd))			
<42.4%	88.1% (266)	79.5% (31)	
>42.4% and <54.6%	7.0% (21)	7.7% (3)	0.13**
>54.6%	5.0% (15)	12.8% (5)	
Clinical indicators of airway inflammation			
Rhinoconjunctivitis-like symptoms||	8.6% (26)	15.4% (6)	0.24**
Asthma-like symptoms¶	3.6% (11)	7.7% (3)	0.21**
Quantitative indicators of airway inflammation			
FENO in ppb (median (first-third quartiles))	12.0 (8.7-16.6)	50.8 (20.4-69.5)	<0.0001††
FENO in percent predicted#			
<140%	93.4% (282)	33.3% (13)	
>140% and <225%	6.6% (20)	12.8% (5)	<0.001**
>225%	0	53.9% (21)	
Eosinophil count in nasal fluid lavage			
<1%	93.1% (281)	33.3% (13)	
>1% and <14%	7.0% (21)	5.1% (2)	<0.001**
>14%	0	61.5% (24)	

Rrs4-16: mean oscillatory resistance from 4 to 16 Hz; MBC Methacholine Bronchial Challenge test.

* asthma or allergy in siblings and/or parents; † hay fever and/or eczema in childhood and/or asthma in childhood; ‡ defined as a positive response to at least one common allergen at the Skin Prick Tests

§ in percent predicted according to the European Respiratory Society

|| in case of itchy, runny, stuffy nose, or sneezes and/or about red, burning or weeping eyes, excepted during a respiratory infection; ¶ in case of wheezing, chest tightness, shortness of breath, or cough, excepted during a respiratory infection or under exercise condition.

# according to gender, tobacco usage status and personal atopy ([13])

** Fisher's exact test; †† Mann-Whitney test

## Discussion

In this study, we have described associations of four markers of airway responsiveness or inflammation and clinical symptoms in a young non-asthmatic population at the start of occupational exposure. We have identified different fragile groups, especially a group with airway eosinophilic inflammation, more frequent BHR and atopy.

As expected, our apprentice population exhibits the characteristics of a healthy population. Mean observed baseline FVC and FEV1 were higher than 90% of predicted values. Prevalence of symptom[18,19] and BHR[19,20] were similar to or lower than those found in other studies of trainees starting apprenticeship and exposed to high weight molecular agents. One study of apprentice bakers revealed a higher prevalence of symptoms[20], but this might be due to the 25% of apprentices who worked in baker shops before starting their training. Prevalence of atopy (32%) was within the range of, or lower than, in other European published studies[18,20,21], whilst higher prevalences (over 40%) were found by Gautrin[19,22], but there is no straightforward explanation for the difference between our study and those of Canadian investigators.

Rhinoconjunctivitis- and asthma-like symptoms were not associated with other markers in our study. Baseline cross-sectional association between symptoms and BHR was not examined in any apprentice study. Two cohort studies failed to find a statistically significant relationship between baseline BHR and incidence of work-related symptoms[23,24]. However, Gautrin found that baseline BHR was significantly associated with development of non-work-related asthma symptoms[19]. Our definition of symptoms was not restricted to work-related symptoms because occupational exposure had just started and it is therefore not surprising that the baseline declared symptoms are not associated with airway inflammation or responsiveness markers.

The fact that the BHR post-MBC test was unrelated to increased AR post methacholine inhalation was unexpected, while it being well known that there is significant correlation between changes in resistance and FEV1 following bronchoconstriction[25]. However, this correlation has been found in workers or in patients, very different from our population of apprentices about length of exposure and health status.

Neither did we discover a relationship between BHR and FENO. Two studies based on community population enrolment have shown that BHR is associated with FENO in atopics only[26,27]. The same atopic-based relationship was found in a population of male conscripts similar to the population used in our study[28].

In our study, BHR proved unrelated to FENO whatever the atopic status (data not shown). However, the study population was effectively different from ours. That is, it featured higher prevalence of atopy (over 50%) and BHR (over 16%) and included asthmatics.

We found that BHR was independent of EP. According to literature, eosinophil numbers in induced sputum is more often used than the same count in nasal lavage fluid and was not significantly associated with BHR in 32 healthy controls used in a comparative study of airway inflammation and epithelial damage in swimmers and cold-air athletes[29].

Based on the combined markers of airway inflammation and hyperresponsiveness, we found that two axes effectively structure the data: one involving airway inflammation and one involving symptoms and AR. We performed a further analysis based on predicted FEV1 percentage and these axes and the item groups remained the same in this analysis [Additional file 4]. This strengthens our conclusion that both FENO and EP are components of our data structure.

Eosinophilic inflammation revealed a statistically significant association with BHR, whilst BHR-FENO and BHR-EP associations were statistically insignificant. This might be due to greater power through an increase in the number of subjects with eosinophilic inflammation either based on FENO or EP.

The relationship between eosinophilic inflammation and increased AR post-MBC test is stronger than between BHR and AR (p = 0.13, p = 0.75 respectively). This was unexpected and, as far as we know, the first time AR and FENO or EP have been analysed together.

We found eosinophilic inflammation to be strongly associated with personal atopy. This is meaningful because atopy is not used to build groups with MCA and hierarchical classifications, whereas FENO, eosinophils and BHR are. The relationship between increased FENO and atopy is well known[9] and an understanding of FENO values requires atopy assessment[13]. Atopy was associated with increased eosinophil counts in induced sputum in 50 healthy subjects[30]. Amongst healthy adults, personal atopy is significantly associated with higher eosinophil counts[31]. In our study, atopy is significantly associated with BHR, post-MBC test AR, FENO, except with EP [Additional file 1]. Combined use of FENO and EP may have proved a more powerful method of revealing an association.

One strength of this study is that it is based on one of the widest European apprentice cohorts. The drop-out is low (20%)[11] and it represents a healthy population at the start of occupational exposure, thereby allowing us to consider early disorders. Another strength is that several markers were collected and analysed together, especially FENO, which studied for the first time among apprentices. On the other hand, a weakness of this study is that it is based on cross-sectional analysis and the clinical significance of the different identified groups is still unknown. Olin et al. have shown that increased FENO is predictive of wheeze 4 years later in a baseline non asthmatic non wheezing general population[10]. Our cohort follow-up has now been completed and we'll seek if our results support her hypothesis that increased FENO is a sign of sub-clinical inflammation in the near future.

## Conclusions

In a young non-asthmatic population at the beginning of occupational exposure, we have identified cross-sectionally different groups defined by their airway inflammation markers. The longitudinal follow up of this population will allow to check whether these groups are predictive of an aggravation.

## Abbreviations

AR: Airway Resistance; BHR: Bronchial Hyperresponsiveness; ECP: Eosinophil Cationic Protein; EI: Eosinophilic Inflammation; EP: Eosinophils Percentages in nasal lavage fluid; FENO: Fractional Exhaled Nitric Oxide; FEV1: Forced Expiratory Volume in 1 second; FVC: Forced Vital Capacity; MBC: Methacholine Bronchial Challenge; MCA: Multiple Correspondence Analysis; OA: Occupational Asthma; Rrs4-16: Resistance from 4 to 16 Hz; Sd: Standard deviation; SPT: Skin Prick Test.

## Competing interests

The authors declare that they have no competing interests.

## Authors'contributions

VD participated in the data acquisition, analysis and interpretation; and was involved in writing the article. PW participated in the study design; data analysis and interpretation; and was involved in writing the article. DZN participated in the study design; was involved in writing the article; and critically reviewed the draft for important intellectual content. PT participated in the data acquisition, analysis and interpretation; and critically reviewed the draft for important intellectual content. AB participated in the study design and in the data acquisition; and critically reviewed the draft for important intellectual content. AB has made substantial contributions to conception and design; and critically reviewed the draft for important intellectual content. CP participated in the data acquisition; was involved in writing the article; and critically reviewed the draft for important intellectual content. All authors have provided approval of the submitted version.

## Pre-publication history

The pre-publication history for this paper can be accessed here:

http://www.biomedcentral.com/1471-2466/10/37/prepub

## Supplementary Material

Additional file 1 **Table One.** Description of associations between atopy and gender, behavioural characteristics and markers of airway responsiveness and inflammation. Click here for file

Additional file 2 **Table Two.** Distribution of absolute FENO values and FENO in percent predicted according to sex, tobacco usage status and atopy. Click here for file

Additional file 3 **Figure One**. Illustration of groups of items resulting from multiple correspondence analysis and hierarchical classification using markers of airway responsiveness and inflammation. Click here for file

Additional file 4 **Figure Two**. Illustration of groups of items resulting from multiple correspondence analysis and hierarchical classification using FEV1 and markers of airway responsiveness and inflammation. Click here for file

## References

[B1] Lemiere C Malo JL Use of induced sputum in the investigation of occupational asthma Med Sci (Paris) 2006 22 595 600 16828034 10.1051/medsci/20062267595

[B2] Zietkowski Z Bodzenta-Lukaszyck A Tomasiak MM Skiepko R Szmitkowski M Comparison of exhaled nitric oxide measurement with conventional tests in steroid-native asthma patients J Investig Allergol Clin Immunol 2006 16 239 246 16889281

[B3] Tufvesson E Aronsson D Ankerst J George SC Bjermer L Peripheral nitric oxide is increased in rhinitis patients with asthma compared to bronchial hyperresponsiveness Respir Med 2007 101 2321 2326 10.1016/j.rmed.2007.06.015 17686621

[B4] Van den Toorn L Prins JB Overbeek SE Hoogsteden HC de Jongste JC Adolescents in clinical remission of atopic asthma have elevated exhaled nitric oxide levels and bronchial hyperresponsiveness Am J Respir Crit Care Med 2000 162 953 957 10988112 10.1164/ajrccm.162.3.9909033

[B5] Polosa R Ciamarra I Mangano G Prosperini G Pistorio MP Vancheri C Crimi N Bronchial hyperresponsiveness and airway inflammatory markers in nonasthmatics with allergic rhinitis Eur Resp J 2000 15 30 35 10.1183/09031936.00.15103000 10678617

[B6] Alvarez MJ Olaguibel JM García BE Rodríquez A Tabar AI Urbiola E Airway inflammation in asthma and perennial allergic rhinitis. Relationship with nonspecific bronchial responsiveness and maximal airway narrowing Allergy 2000 55 355 362 10.1034/j.1398-9995.2000.00312.x 10782520

[B7] Ciprandi G Vizzaccaro A Cirillo I Tosca M Massolo A Passalacqua G Nasal eosinophils display the best correlation with symptoms, pulmonary function and inflammation in allergic rhinitis Int Arch Allergy Immunol 2005 136 266 272 10.1159/000083953 15722636

[B8] Howarth P Persson C Meltzer E Jacobson MR Durham SR Silkoff PE Objective monitoring of nasal airway inflammation rhinitis J Allergy Clin Immunol 2005 115 S414 S441 10.1016/j.jaci.2004.12.1134 15746881

[B9] ATS/ERS Recommendations for Standardized Procedures for the Online and Offline Measurement of Exhaled Lower Respiratory Nitric Oxide and Nasal Nitric Oxide Am J Respir Crit Care Med 2005 171 912 930 10.1164/rccm.200406-710ST 15817806

[B10] Olin AC Rosengren A Thelle DS Lissner L Toren K Increased fraction of exhaled nitric oxide predicts new-onset wheeze in a general population Am J Respir Crit Care Med 2010 181 327 327 10.1164/rccm.200907-1079OC 20007926

[B11] Tossa P Bohadana AB Demange V Wild P Michaely JP Hannhart B Paris C Zmirou-Navier D Early markers of airways inflammation and occupational asthma: Rationale, study design and follow-up rates among bakery, pastry and hairdressing apprentices BMC Public Health 2009 9 113 10.1186/1471-2458-9-113 19389222 PMC2681469

[B12] Minette A Questionnaire of the European Community for Coal and Steel (ECSC) on respiratory symptoms. 1987--updating of the 1962 and 1967 questionnaires for studying chronic bronchitis and emphysema Eur Respir J 1989 2 165 177 2703044

[B13] Travers J Marsh S Aldington S Williams M Shirtcliffe P Pritchard A Weatherall M Beasley R Reference Ranges for Exhaled Nitric Oxide Derived from a Random Community Survey of Adults Am J Respir Crit Care Med 2007 176 238 242 10.1164/rccm.200609-1346OC 17478616

[B14] Quanjer PH Tammeling GJ Cotes JE Pedersen OF Peslin R Yernault JC Lung volumes and forced respiratory flows. Report working party. Standardization of Lung Function Tests. European Community for Coal and Steel Eur Respir J 1993 6 5 40 8499054

[B15] Massin N Bohadana AB Wild P Goutet P Kirstetter H Toamain JP Airway responsiveness, respiratory symptoms, and exposures to soluble oil mist in mechanical workers Occup Environ Med 1996 53 748 752 10.1136/oem.53.11.748 9038798 PMC1128592

[B16] Hilding AC Simple method for collecting near-normal human nasal secretion Ann Otol Rhinol Laryngol 1972 81 422 423 5030765 10.1177/000348947208100314

[B17] Maria Y Moneret-Vautrin DA Pham QT Teculescu D Bouchy O Chau N Lamaze C Adrian E Tagu P Sensibilisation cutanée aux allergènes « respiratoires » chez les exploitants et salariés agricoles Rev Mal Respir 1991 8 463 471 1767118

[B18] Walusiak J Palczynski C Hanke W Wittczak T Krakowiak A Górski P The risk factors of occupational hypersensitivity in apprentice bakers - the predictive value of atopy markers Int Arch Occup Environ Health 2002 75 S117 S121 10.1007/s00420-002-0358-9 12397422

[B19] Gautrin D Ghezzo H Malo JL Rhinoconjunctivitis, bronchial responsiveness, and atopy as determinants for incident non-work-related asthma symptoms in apprentices exposed to high-molecular-weight allergens Allergy 2003 58 608 615 10.1034/j.1398-9995.2003.00197.x 12823119

[B20] Skjold T Nielsen SC Adolf K Hoffmann HJ Dahl R Sigsgaard T Allergy in bakers' apprentices and factors associated to non-participation in a cohort study of allergic sensitization Int Arch Occup Environ Health 2007 80 458 464 10.1007/s00420-006-0151-2 17021842

[B21] Talini D Monteverdi A Lastrucci L Buonocore C Carrara M Di Pede F Paggiaro P One-year longitudinal study of young apprentices exposed to airway occupational sensitizers Int Arch Occup Environ Health 2006 79 237 243 10.1007/s00420-005-0040-0 16235084

[B22] Monso E Malo JL Infante-Rivard C Ghezzo H Magnan M L'Archevèque J Trudeau C Gautrin D Individual characteristics and quitting in apprentices exposed to high-molecular-weight agents Am J Respir Crit Care Med 2000 161 1508 1512 10806146 10.1164/ajrccm.161.5.9906113

[B23] Skjold T Dahl R Juhl B Sigsgaard T The incidence of respiratory symptoms and sensitisation in baker apprentices Eur Respir J 2008 32 452 459 10.1183/09031936.00108207 18417513

[B24] Gautrin D Ghezzo H Infante-Rivard C Malo JL Incidence and host determinants of work-related rhinoconjunctivitis in apprentice pastry-makers Allergy 2002 57 913 918 10.1034/j.1398-9995.2002.23636.x 12269937

[B25] Oostveen E MacLeod D Lorino H Farré R Hantos Z Desager K Marchal F ERS task force The forced oscillation technique in clinical practice: methodology, recommendations and future developments Eur Respir J 2003 22 1026 1041 10.1183/09031936.03.00089403 14680096

[B26] Franklin PJ Turner SW Le Souëf PN Stick SM Exhaled nitric oxide and asthma: complex interactions between atopy, airway responsiveness, and symptoms in a community population of children Thorax 2003 58 1048 1052 10.1136/thorax.58.12.1048 14645971 PMC1746531

[B27] Franklin PJ Stick SM Le Souëf PN Ayres JG Turner SW Measuring exhaled nitric oxide levels in adults: the importance of atopy and airway responsiveness Chest 2004 126 1540 1545 10.1378/chest.126.5.1540 15539724

[B28] Rouhos A Ekroos H Karjalainen J Sarna S Sovijärvi AR Exhaled nitric oxide and exercise-induced bronchoconstriction in young male conscripts: association only in atopics Allergy 2005 60 1493 1498 10.1111/j.1398-9995.2005.00901.x 16266380

[B29] Bougault V Turmel J St-Laurent J Bertrand M Boulet LP Asthma, airway inflammation and epithelial damage in swimmers and cold-air athletes Eur Respir J 2009 33 713 714 10.1183/09031936.00117708 19129276

[B30] Berlyne GS Parameswaran K Kamada D Efthimiadis A Hargreave FE A comparison of exhaled nitric oxide and induced sputum as markers of airway inflammation J Allergy Clin Immunol 2000 106 638 644 10.1067/mai.2000.109622 11031333

[B31] Belda J Leigh R Parameswaran K O'Byrne PM Sears MR Hargreave FE Induced sputum cell counts in healthy adults Am J Respir Crit Care Med 2000 161 475 478 10673188 10.1164/ajrccm.161.2.9903097

