# Supplementary material for: Associations of airway inflammation and responsiveness markers in non asthmatic subjects at start of apprenticeship
Source: BMC Pulm Med. 2010 Jul 6;10:37. doi: 10.1186/1471-2466-10-37 (PMC2913998; doi:10.1186/1471-2466-10-37)
Supplement: Additional file 1 — Table One. Description of associations between atopy and gender, behavioural characteristics and markers of airway responsiveness and inflammation. [file 1471-2466-10-37-S1.DOC]

Table 1. Associations between atopy defined on the basis of prick tests and gender, behavioural characteristics and markers of airway responsiveness and inflammation (percentage in column (n)) (N=280).

|  | Non atopics  (N=197) | Atopics  (N=83) | p |
| --- | --- | --- | --- |
| Constitutional characteristics |  |  |  |
| Gender: Male versus Female | 52 (102)/48 (95) | 65 (54)/35 (29) | 0.04 |
| Atopy in family* | 39.1 (77) | 38.6 (32) | 0.93 |
| Clinical atopy† | 16.2 (32) | 24.1 (20) | 0.12 |
| Behavioural characteristics |  |  |  |
| Training track |  |  |  |
| Bakery | 32.5 (64) | 43.4 (36) | 0.08 |
| Pastry making | 24.9 (49) | 27.7 (23) |
| Hairdressing | 42.6 (84) | 28.9 (24) |
| Tobacco status |  |  |  |
| Non smoker | 48.7 (96) | 45.8 (38) | 0.92# |
| Current smoker | 47.7 (94) | 50.6 (42) |
| Past smoker | 3.6 (7) | 3.6 (3) |
| Baseline FEV1‡ |  |  |  |
| >80.8% | 88.8% (175) | 83.1% (69) |  |
| >76.0% and < 80.7% | 7.1% (14) | 9.6% (8) | 0.31# |
| <76.0% | 4.1% (8) | 7.2% (6) |  |
| MBC test |  |  |  |
| Airways responsiveness |  |  |  |
| MBC- | 89.9 (177) | 74.7 (62) | 0.002# |
| MBC+ ≥15 and < 20% (% (n)) | 7.1 (14) | 12.1 (10) |
| MBC+ ≥20% (% (n)) | 3.1 (6) | 13.3 (11) |
| Rrs4-16 increase (%) (mean (sd)) |  |  |  |
| <42.4% | 89.9 (177) | 78.3 (65) | 0.006# |
| > 42.4% and <54.6% | 6.6 (13) | 7.2 (6) |
| > 54.6% | 3.6 (7) | 14.5 (12) |
| Clinical indicators of airway inflammation |  |  |  |
| Rhinoconjunctivitis-like symptoms§ | 11.2 (22) | 10.8 (9) | 0.94 |
| Asthma-like symptoms║ | 4.1 (8) | 4.8 (4) | 0.75# |
| Quantitative indicators of airway inflammation |  |  |  |
| FENO in ppb (median (first-third quartiles)) | 11.2 (8.2-15.3) | 17.2 (11.2-27.9) | <0.0001 |
| FENO in percent predicted¶ |  |  |  |
| <140% | 93.4 (184) | 74.7 (62) | <0.001# |
| >140% and <225% | 4.6 (9) | 13.3 (11) |
| >225% | 2.0 (4) | 12.1 (10) |
| Eosinophil count in nasal fluid lavage |  |  |  |
| <1% | 88.8 (175) | 80.7 (67) | 0.15 |
| >1% and <14% | 6.1 (12) | 8.4 (7) |
| >14% | 5.1 (10) | 10.8 (9) |

* asthma or allergy in siblings and/or parents; † hay fever and/or eczema in childhood and/or asthma in childhood.

‡ in percent predicted according to the European Respiratory Society

§ in case of itchy, runny, stuffy nose, or sneezes and/or about red, burning or weeping eyes, excepted during a respiratory infection; ║ in case of wheezing, chest tightness, shortness of breath, or cough, excepted during a respiratory infection or under exercise condition.

¶ according to gender, tobacco usage status and personal atopy ([13])

# Fisher’s exact test
