# Supplementary material for: Associations of airway inflammation and responsiveness markers in non asthmatic subjects at start of apprenticeship
Source: BMC Pulm Med. 2010 Jul 6;10:37. doi: 10.1186/1471-2466-10-37 (PMC2913998; doi:10.1186/1471-2466-10-37)
Supplement: Additional file 2 — Table Two. Distribution of absolute FENO values and FENO in percent predicted according to sex, tobacco usage status and atopy. [file 1471-2466-10-37-S2.DOC]

Table 2. Distribution of absolute FENO values and FENO in percent predicted with cutoffs used in analyses according to sex, tobacco usage status and atopy (a positive response to at least one common allergen at the Skin Prick Tests).

| In ppb | <25 ppb | | | | | | 25-45 ppb | | | | >45 ppb | | |
| --- | --- | --- | --- | --- | --- | --- | --- | --- | --- | --- | --- | --- | --- |
| In % predicted | <140% | 140%-225% | | | >225% | | <140% | 140%-225% | | >225% | <140% | 140%-225% | >225% |
| Sex |  |  | | |  | |  |  | |  |  |  |  |
| Males | N=186 | | | | | | N=25 | | | | N=18 | | |
|  | 186 (100%) | | | 0 | | 0 | 8 (32%) | | 17 (68%) | 0 | 0 | 3 (17%) | 15 (83%) |
| Females | N=139 | | | | | | N=13 | | | | N=10 | | |
|  | 136 (98%) | | 3 (2%) | | | 0 | 2 (15%) | | 10 (77%) | 1 (8%) | 0 | 0 | 10 (100%) |
| Tobacco usage status |  | |  | | |  |  | |  |  |  |  |  |
| Non smoker | N=154 | | | | | | N=21 | | | | N=19 | | |
|  | 153 (99%) | | 1 (1%) | | | 0 | 7 (33%) | | 14 (67%) | 0 | 0 | 3(16%) | 16 (84%) |
| Current smoker | N=159 | | | | | | N=17 | | | | N=7 | | |
|  | 157 (99%) | | 2 (1%) | | | 0 | 3 (18%) | | 13 (76%) | 1 (6%) | 0 | 0 | 7 (100%) |
| Past smoker | N=12 | | | | | | N=0 | | | | N=2 | | |
|  | 12 (100%) | | 0 | | | 0 | 0 | 0 | | 0 | 0 | 0 | 2 (100%) |
| Personal atopy | N=61 | | | | | | N=19 | | | | N=15 | | |
| Yes | 61 (100%) | | 0 | | | 0 | 8 (42%) | | 11 (58%) | 0 | 0 | 2 (13%) | 13 (87%) |
| No | N=205 | | | | | | N=10 | | | | N=3 | | |
|  | 203 (99%) | | 2 (1%) | | | 0 | 0 | 9 (90%) | | 1 (10%) | 0 | 0 | 3 (100%) |
