# Supplementary material for: Associations of airway inflammation and responsiveness markers in non asthmatic subjects at start of apprenticeship
Source: BMC Pulm Med. 2010 Jul 6;10:37. doi: 10.1186/1471-2466-10-37 (PMC2913998; doi:10.1186/1471-2466-10-37)
Supplement: Additional file 3 — Figure One. Illustration of groups of items resulting from multiple correspondence analysis and hierarchical classification using markers of airway responsiveness and inflammation. [file 1471-2466-10-37-S3.DOC]

Group 1

Group 2

Group 3

Group 4

0

1

2

0

1

2

0

1

2

0

1

2

0

1

0

1

-4

-2

0

2

4

6

-6

-4

-2

0

2

dimension 1 (57.9%)

coordinates in standard normalization

MCA coordinate plot

FENO
 BHR

% eosinophils
 Resistance

Rhinoconjunct.
 Asthma-like

dimension 2 ( 9.1%)

2: highest levels in FENO, eosinophils percentages, MCT+, airway resistance; 1: middle levels in FENO, eosinophils percentages, MCT+, airway resistance or presence of rhinoconjunctivitis-like symptoms and asthma-like symptoms; 0: lowest levels in FENO, eosinophils percentages, airway resistance or negative MCT test or absence of symptoms

Figure 1. Groups of items resulting from multiple correspondence analysis and hierarchical classification using FENO (Travers predicted values [13]), bronchial hyperresponsiveness, increase in airway resistance post methacholine, eosinophils percentages in nasal lavage fluid and rhinoconjunctivitis-like symptoms and asthma-like symptoms.

Group 1: highest levels in FENO and/or eosinophils percentages.

Group 2: highest levels in MCT+ and/or middle level in eosinophils percentages.

Group 3: highest level in increase of airway resistance and/or middle levels in MCT+, FENO and/or existence of rhinoconjunctivitis-like and/or asthma-like symptoms.

Group 4: negative MCT test and/or lowest values of FENO and/or lowest values in eosinophils percentages and/or lowest or middle values of airway resistance and/or no symptoms.
