# Supplementary material for: Associations of airway inflammation and responsiveness markers in non asthmatic subjects at start of apprenticeship
Source: BMC Pulm Med. 2010 Jul 6;10:37. doi: 10.1186/1471-2466-10-37 (PMC2913998; doi:10.1186/1471-2466-10-37)
Supplement: Additional file 4 — Figure Two. Illustration of groups of items resulting from multiple correspondence analysis and hierarchical classification using FEV1 and markers of airway responsiveness and inflammation. [file 1471-2466-10-37-S4.DOC]

Group 1

Group 2

Group 3

Group 4

0

1

2

0

1

2

0

1

2

0

1

2

0

1

0

1

0

1

2

-5

0

5

10

dimension 2 (10.5%)

-6

-4

-2

0

2

dimension 1 (50.1%)

coordinates in standard normalization

MCA coordinate plot

FENO

BHR

% eosinophils

Resistance

Rhinoconjunct.

Asthma-like

FEV1 % predicted

2: highest levels in FENO, eosinophils percentages, MCT+, airway resistance, lowest level in FEV1 % predicted; 1: middle levels in FENO, eosinophils percentages, MCT+, FEV1 % predicted, airway resistance or presence of rhinoconjunctivitis-like symptoms and asthma-like symptoms; 0: lowest levels in FENO, eosinophils percentages, airway resistance or negative MCT test or highest FEV1 % predicted or absence of symptoms

Figure 2. Groups of items resulting from multiple correspondence analysis and hierarchical classification using FEV1 in percent predicted ([14]), FENO (Travers predicted values [13]), bronchial hyperresponsiveness, increase in airway resistance post methacholine, eosinophils percentages in nasal lavage fluid and rhinoconjunctivitis-like symptoms and asthma-like symptoms.

Group 1: highest levels in FENO and/or eosinophils percentages and/or middle level in MCT+.

Group 2: highest levels in MCT+ and/or middle level in eosinophils percentages and/or lowest level of FEV1 in percent predicted.

Group 3: existence of asthma-like symptoms.

Group 4: negative MCT test and/or middle or lowest values of FENO and/or lowest values in eosinophils percentages and/or airway resistance (whatever level) and/or rhinoconjunctivitis-like symptoms and/or no symptom.
